# Risk factors for serotype 19A carriage after introduction of 7-valent pneumococcal vaccination

**DOI:** 10.1186/1471-2334-11-95

**Published:** 2011-04-18

**Authors:** Robert Cohen, Corinne Levy, Eric Bonnet, Franck Thollot, Michel Boucherat, Bernard Fritzell, Véronique Derkx, Edouard Bingen, Emmanuelle Varon

**Affiliations:** 1 Department of Microbiology, CHI Créteil 40 Avenue de Verdun, France; 2 ACTIV, Association Clinique Thérapeutique Infantile du Val de Marne, 27 rue Inkermann F94100 Saint Maur des Fossés, France; 3 23 av Doct Lannelongue 75014 PARIS, Laboratoire Pfizer, France; 4 AFPA, Association Française de Pédiatrie Ambulatoire 4 rue Parmentier F54270 Essey les Nancy, France; 5 Department of Microbiology, Université Denis-Diderot-Paris7, Robert Debré hospital (AP-HP), 48 Bd Sérurier 75019 Paris, France; 6 National Reference Center for Pneumococci, AP-HP, HEGP 20, rue Leblanc, F75015 Paris, France

## Abstract

**Background:**

After the implementation of 7-valent pneumococcal conjugate vaccine (PCV7), in several countries, serotype 19A is now the serotype most frequently involved in pneumococcal diseases and carriage. To determine factors potentially related to 19A nasopharyngeal (NP) carriage we analyzed data from an ongoing prospective French national surveillance study of pneumococcal NP carriage in young children.

**Methods:**

NP swabs were obtained from children aged 6 to 24 months, either during routine check-ups with normal findings, or when they presented with acute otitis media (AOM). The swabs were sent for analysis to the French National Reference Centre for Pneumococci. Factors influencing pneumococcal carriage and carriage of penicillin non-susceptible (PNSP), 19A and PNS-19A were investigated by multivariate logistic regression.

**Results:**

From 2006 to 2009, 66 practitioners enrolled 3507 children (mean age 13.6 months), of whom, 98.3% of children had been vaccinated with PCV7 and 33.4% of children attended daycare centres (DCC). Serotype 19A was found in 10.4% of the overall population, 20.5% of *S. pneumoniae *carriers (n = 1780) and 40.8% of PNSP carriers (n = 799). Among 19A strains, 10.7% were penicillin-susceptible, 80% intermediate and 9.3% fully resistant. Logistic regression analysis showed that the main factors associated with PNSP carriage were AOM (OR = 3.09, 95% CI [2.39;3.98]), DCC (OR = 1.70, 95% CI [1.42;2.03]), and recent antibiotic use (OR = 1.24, 95% CI [1.05;1.47]. The main factors predictive of 19A carriage were recent antibiotic use (OR = 1.81, 95% CI [1.42;2.30]), AOM (OR = 1.67, 95% CI [1.11;2.49]), DCC (OR = 1.56, 95% CI [1.21;2.2] and young age, <12 months (OR = 1.51, 95% CI [1.16;1.97]).

**Conclusion:**

In a population of children aged from 6 to 24 months with a high rate of PCV7 vaccination coverage, we found that antibiotic exposure, DCC attendance and AOM were linked to 19A carriage.

## Background

An increase in the incidence of *S. pneumoniae *serotype 19A isolation has been observed in many countries, and 19A is now the serotype most frequently isolated from patients with invasive and mucosal pneumococcal diseases [1-4]. Serotype 19A is also a frequent nasopharyngeal (NP) carriage serotype, and is frequently resistant to antibiotics [3,5,6]. Most non-PCV7 serotypes do not share all of these properties, and it is important to identify factors determining carriage serotype patterns [7]. In France, the increasing involvement of serotype 19A in NP carriage, AOM and invasive disease is related to clonal expansion of the pre-existing penicillin-intermediate ST 276, [5,8] whereas in the US the increase in serotype 19A is mainly attributed to ST199 and the multidrug-resistant clone ST 320 [4]. Vaccine escape was first reported in the US by Brueggeman et al. Around 2003, recombination occurred between the recipient ST695 serotype 4 and donor ST199 serotype 19A, simultaneously resulting in the non vaccine capsular type and penicillin intermediate-resistant ST695 19A pneumococci. With the additional selective advantage of penicillin non susceptibility, the ST695 19A variant continued to spread, becoming in 2007 the fourth most common serotype 19A clonal complex in the US [9]. In several countries, the prevalence of 19A started to rise after implementation of 7-valent pneumococcal conjugate vaccine (PCV7) [4,10] but in Israel and South Korea an increase was noted before the vaccine era, and the observed association with antibiotic use pointed to a role of secular variations [11,12]. The NP flora is the main ecological niche of *S. pneumoniae *(Sp), and the onset of invasive and non invasive diseases is often preceded by carriage acquisition [13,14]. The marked decline in vaccine serotypes observed since PCV7 was implemented has made room for non vaccine serotypes (NVS) including 19A. We therefore examined factors potentially related to 19A NP carriage by analyzing data from an ongoing prospective French national surveillance study of pneumococcal NP carriage in young children [8,15].

## Methods

From November 2006 to June 2009, 66 French pediatricians and general practitioners distributed throughout France took part in a cross-sectional study. An NP swab was obtained from children aged 6 to 24 months, either during routine check-ups with normal findings, or when they presented with AOM. Children were excluded from the study if they had received antibiotics within 7 days before enrolment, or had a severe underlying health disorder, or had already been included in the study during the previous 12 months. Demographic data, the medical history and physical findings were recorded. The study was approved by the Saint Germain en Laye Ethics Committee, and written informed consent was obtained from the parents or guardians. Nasopharyngeal specimens were obtained with cotton-tipped wire swabs. The swabs were inserted into the anterior nares, gently rubbed on the nasopharyngeal wall and immediately placed in transport medium (Copan Venturi Transystem^®^, Brescia, Italy). The samples were transferred within 48 hours to the French National Reference Centre for Pneumococci. *S. pneumoniae *culture, identification, serotyping and antibiotic susceptibility testing were performed as previously described [15]. Susceptibility of *S. pneumoniae *isolates to penicillin G was determined from minimal inhibitory concentrations (MICs) by the agar-dilution method. Isolates were classified as penicillin-susceptible (MIC ≤ 0.06 μg/ml), intermediate (0.12 ≤ MIC ≤ 1.0 μg/ml) or fully resistant (MIC ≥ 2 μg/ml) as recommended by the Antibiogram Committee of the French Society of Microbiology [8,15,16].

Data were double-entered using 4D software (version 6.4), and analysed using Stata SE 9.1 (Stata Corp., College Station, TX, USA) for univariate analysis and multivariate logistic regression (odds ratios [ORs] and 95% confidence intervals [CI]). The Pearson Chi-square test was used in univariate analysis to identify factors related (P < 0.10) to NP carriage of pneumococci, serotype 19A, and penicillin non-susceptible pneumococci (PNSP = intermediate + resistant: MIC ≥ 0.12 μg/ml) [16]. 19A serotype and penicillin non susceptible 19A serotype (PNS-19A) carriage was also separately analysed among Sp and PNSP carriers, respectively. Variables identified by univariate analysis were age, gender, the study group (healthy controls vs children with AOM), PCV7 vaccination status, the season, year of enrolment, existence of siblings, daycare attendance, recent antibiotic treatment (within 3 months before enrolment), and nasopharyngeal carriage of *H. influenzae*, *M. catarrhalis *and *S. aureus*. Winter months were November to March and spring months April to June. PCV7 vaccination was considered partial when the primary series of immunization as recommended in France was incomplete in children < 12 months or when no booster dose had been administered to children ≥12 months, and complete if all initial injections had been received by age 12 months, and if the booster dose had been administered to children ≥12 months old.

## Results

**Figure 1:**
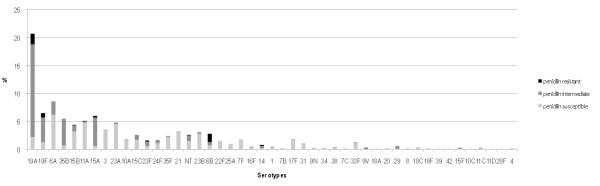
**Serotypes distribution among the pneumococcal carriers according to penicillin resistance**.

Sixty-six practitioners enrolled 3507 patients (909 healthy controls and 2598 children with AOM). Table 1 shows demographic characteristics and nasopharyngeal carriage of the children enrolled. Mean age was 13.6 ± 5.2 months (median 12.8) and 98.3% of children had been vaccinated with PCV7 (partial 21.9%, complete 78.1%). One-third (33.4%) of children attended daycare centres (DCC), of whom 45.3% had siblings, while 34.5% of children were kept at home, of whom 68.9% had siblings. The pneumococcal carriage rate was 50.8% in the overall population. The isolates were penicillin-susceptible in 54.9% of cases, intermediate in 41.3% and fully resistant in 3.8%. Figure 1 shows the serotype distribution and the resistance profile among pneumococcal carriers according to the serotype. Serotype 19A was clearly the leading serotype (20.5% of isolates). All PCV7 vaccine types represented less than 11%, serotypes 15A, 15B and 15C respectively 6.1%, 4.3% and 2.6%, and serotype 35B, 5.5%. Among 19A strains, 10.7% were penicillin-susceptible, 80% intermediate and 9.3% fully resistant. Serotype 19A represented 40.8% of PNSP (326/799). The other non vaccine PNSP serotypes were 35B (10.7%) and 15A (12.2%). Table 2 lists factors influencing pneumococcal carriage and carriage of PNSP, 19A and PNS-19A in logistic regression analysis. Logistic regression analysis showed that the main factors associated with PNSP carriage were AOM (OR = 3.09, 95% CI [2.39;3.98]), DCC (OR = 1.70, 95% CI [1.42;2.03]), and recent antibiotic use (OR = 1.24, 95% CI [1.05;1.47]. Whatever the analysis, logistic regression confirmed that AOM, DCC, recent antibiotic use and young age (<12 months) were predictive of 19A carriage. Sibling status increased the risk of Sp carriage but reduced the risk of 19A carriage.

**Table 1:** Demographic characteristics and nasopharyngeal carriage in children with AOM and healthy controls

	Total N = 3507 n (%)	Healthy controls N = 909 Total n (%)	Children with AOM N = 2598 Total n (%)	p
**Demographic characteristics**				
Sex M	1818 (51.8)	442 (48.6)	1376 (53)	0.02
Mean age ± SD (months)	13.6 ± 5.2	13.1 ± 5.4	13.8 ± 5.1	0.0008
Vaccination with PCV7*	3448 (98.3)	890 (97.9)	2558 (98.5)	0.2
Partial	754 (21.9)	149 (16.7)	605 (23.7)	
Complete	2693 (78.1)	741 (83.3)	1952 (76.3)	
Type of care				<0.0001
Day care centre	1170 (33.4)	138 (15.2)	1032 (39.7)	
Childminder	1127 (32.1)	316 (34.8)	811 (31.2)	
Home	1209 (34.5)	455 (50.1)	754 (29.1)	
Siblings	1906 (54.3)	420 (46.2)	1486 (57.2)	<0.0001
Use of Antibiotics 3 months before	1439 (41.0)	195 (21.5)	1244 (47.9)	<0.0001

**Nasopharyngeal carriage**				

***S. pneumoniae***	1780 (50.8)	270 (29.7)	1510 (58.1)	<0.0001
Penicillin susceptibility**				0.0001
Susceptible	974 (54.9)	182 (68.2)	792 (52.6)	
Intermediate	732 (41.3)	76 (28.5)	656 (43.5)	
Resistant	67 (3.8)	9 (3.3)	58 (3.9)	

***Haemophilus influenzae***	1399 (39.9)	130 (14.3)	1269 (48.8)	<0.0001

***Moraxella catarrhalis***	1780 (50.8)	301 (33.1)	1479 (56.9)	<0.0001

***Staphylococcus aureus***	235 (6.7)	99 (10.9)	136 (5.2)	<0.0001

Multiple carriage (ie at least two species)	1715 (48.9)	213 (23.4)	1502 (57.8)	<0.0001

No bacteria identified	563 (16.1)	376 (41.4)	187 (7.2)	<0.0001

* missing data for PCV7 vaccination status: n = 1

**missing data for penicillin susceptibility: n = 7

**Table 2:** Risk factors for carriage of *S. pneumoniae*, penicillin non susceptible *S. pneumoniae *(PNSP), serotype 19A, and penicillin non susceptible (PNS) serotype 19A

	Univariate analysis			Multivariate analysis		
	OR	[95% CI]	P	OR	[95% CI]	P
**Carriage of Sp (n = 1780)**						
AOM	3.28	2.79;3.86	<0.0001	**2.98**	**2.49;3.56**	**<0.0001**
Day care centre	1.37	1.26;1.48	<0.0001	**1.55**	**1.32;1.82**	**<0.0001**
Siblings	1.58	1.39;1.81	<0.0001	**1.63**	**1.41;1.89**	**<0.0001**
Carriage of *M. catarrhalis*	1.83	1.60;2.09	<0.0001	**1.46**	**1.27;1.69**	**<0.0001**
Age <12 months	1.02	0.89;1.16	0.78	1.12	0.96;1.30	0.16
Carriage of *H. influenzae*	1.24	1.08;1.41	0.002	**0.81**	**0.69;0.94**	**0.006**
Recent use of antibiotic	1.07	0.94;1.23	0.31	**0.75**	**0.65;0.87**	**<0.0001**
Carriage of *S. aureus*	0.50	0.38;0.66	<0.0001	**0.62**	**0.46;0.83**	**0.001**
Partial vaccination	1.28	0.75;2.18	0.37	1.25	0.70;2.20	0.45

**Carriage of PNSP (n = 799)**						
AOM	3.67	2.89;4.66	<0.0001	**3.09**	**2.39;3.98**	**<0.0001**
Day care centre	1.52	1.38;1.68	<0.0001	**1.70**	**1.42;2.03**	**<0.0001**
Siblings	1.06	0.90;1.24	0.5	1.07	0.91;1.27	0.8
Carriage of *M. catarrhalis*	1.51	1.29;1.77	<0.0001	1.15	0.97;1.37	0.09
Age <12 months	1.06	0.90;1.24	0.5	**1.24**	**1.04;1.49**	**0.018**
Carriage of *H. influenzae*	1.18	1.00;1.38	0.043	**0.82**	**0.69;0.97**	**0.002**
Recent use of antibiotic	1.63	1.39;1.91	<0.0001	**1.24**	**1.05;1.47**	**0.011**
Carriage of *S. aureus*	0.72	0.51;1.02	0.062	0.90	0.63;1.29	0.57
Partial vaccination	1.69	0.84;3.40	0.142	1.56	0.76;3.22	0.25

**Carriage of serotype 19A (n = 365) in overall population (n = 3507)**						
AOM	3.89	2.70;5.61	<0.0001	**3.26**	**2.22;4.80**	**<0.0001**
Day care centre	1.63	1.42;1.87	<0.0001	**1.85**	**1.46;2.35**	**<0.0001**
Siblings	0.73	0.58;0.90	0.004	**0.74**	**0.59;0.93**	**0.009**
Carriage of *M. catarrhalis*	1.50	1.20;1.87	<0.0001	1.10	0.87;1.39	0.40
Age <12 months	1.20	0.97;1.50	0.09	**1.48**	**1.16;1.90**	**0.002**
Carriage of *H. influenzae*	1.02	0.81;1.27	0.88	**0.73**	**0.57;0.92**	**0.008**
Recent use of antibiotic	1.92	1.55;2.3	<0.0001	**1.49**	**1.19;1.88**	**0.001**
Carriage of *S. aureus*	0.66	0.39;1.08	0.10	0.80	0.48;1.35	0.41
Partial vaccination	3.81	0.91;15.89	0.066	3.66	0.86;15.49	0.08

**Carriage of serotype 19A (n = 365) among Sp carriers (n = 1780)**						
AOM	2.02	1.38;2.97	0.012	**1.67**	**1.11;2.49**	**0.012**
Day care centre	1.41	1.22;1.63	0.001	**1.56**	**1.21;2.02**	**0.001**
Siblings	0.52	0.41;0.65	<0.0001	**0.56**	**0.44;0.71**	**<0.0001**
Carriage of *M. catarrhalis*	1.09	0.86;1.37	0.49	0.96	0.75;1.22	0.73
Age <12 months	1.22	0.97;1.53	0.09	**1.51**	**1.16;1.97**	**0.003**
Carriage of *H. influenzae*	0.89	0.71;1.13	0.34	0.83	0.65;1.07	0.15
Recent use of antibiotic	2.00	1.59;2.53	<0.0001	**1.81**	**1.42;2.30**	**<0.0001**
Carriage of *S. aureus*	1.02	0.59;1.77	0.93	1.11	0.63;1.96	0.37
Partial vaccination	3.69	0.86;15.85	0.079	3.57	0.81;15.80	0.09

**Carriage of PNS serotype 19A (n = 326) among PNSP carriers (n = 799)**						
AOM	1.89	1.15;3.10	0.012	1.55	0.92;2.61	0.09
Day care centre	1.29	1.09;1.54	0.004	**1.39**	**1.01;1.89**	**0.04**
Siblings	0.52	0.39;0.70	<0.0001	**0.57**	**0.43;0.77**	**<0.0001**
Carriage of *M. catarrhalis*	1.04	0.78;1.39	0.79	0.96	0.71;1.30	0.77
Age <12 months	1.17	0.88;1.55	0.29	1.29	0.93;1.79	0.13
Carriage of *H. influenzae*	0.86	0.64;1.14	0.3	0.80	0.59;1.09	0.16
Recent use of antibiotic	1.59	1.20;2.11	0.001	**1.46**	**1.08;1.97**	**0.013**
Carriage of *S. aureus*	0.79	0.41;1.50	0.46	0.86	0.44;1.68	0.66
Partial vaccination	2.72	0.56;13.16	0.21	2.56	0.51;12.94	0.3

## Discussion

In multiple surveillance studies, serotype 19A strains have been identified as major replacement strains in pneumococcal disease (invasive disease and AOM) reflecting both their greater predominance in carriage and their disease potential. However, the importance of 19A as a replacement serotype in invasive diseases, AOM and NP carriage varies across countries where PCV7 has been implemented, for reasons that are not fully understood [5,17,18]. Previous studies have identified two important factors affecting serotype 19A carriage, namely the prevalence prior to PCV7 implementation, and the level of antibiotic use (a large proportion of 19A strains were non susceptible prior to vaccine implementation) [19]. However, non susceptible strains predominated among other serotypes that have expanded to a lesser extent [19]. Here we investigated other potential risk factors for 19A carriage.

In our population of children aged from 6 to 24 months with a high rate of PCV7 vaccine coverage, we confirmed the importance of antibiotic exposure, and also found a role of DCC attendance and AOM.

Several studies have shown that DCC attendance increases the risk of pneumococcal carriage [8,20]. However, this is the first time that DCC attendance has been specifically linked to increased 19A carriage (OR = 1.85, 95% CI [1.46-;2.35]). The explanation is not only that DCC attendance increased the overall risk of pneumococcal colonization, and 19A was the most frequently carried serotype in this population of highly PCV7-vaccinated children. In addition, 19A was the most prevalent serotype in the subpopulation of DCC attendees carrying pneumococci (OR = 1.56, 95% CI [1.21-;2.02]). The fact that 19A is frequently resistant to antibiotics and that DCC attendees have higher antibiotic exposure does not fully explain our observations. Indeed, 19A carriage was also the most prevalent serotype among carriers of resistant strains (OR = 1.39, 95% CI [1.01-;1.89]). The proportion of children attending DCC in a given country may thus influence the observed increase in 19A disease.

Children with AOM were also more likely to carry serotype 19A. As 19A has already shown to be the most frequent middle-ear-fluid isolate in PCV7-vaccinated children, this result might have been expected. However, our results also support the hypothesis that 19A has higher AOM disease potential than other non vaccine serotypes [6].

The main limitation of our study is the homogeneity of our population (children aged 6 to 24 months, PCV7-vaccinated, without antibiotics exposure within 7 days before enrolment, and with no severe underlying health disorders) making it more difficult to extrapolate our findings to other populations. Furthermore, our study is based on nasopharyngeal carriage results and cannot be directly extrapolated to invasive pneumococcal disease or middle-ear infections. Future implementation of a pneumococcal conjugate vaccine that includes a 19A conjugate may resolve the problems posed by serotype 19A, provided such a vaccine has a significant impact on 19A carriage. Ongoing surveillance of carriage is therefore necessary, because the primary pneumococcal reservoir is the nasopharynx of children. Hence, early insight into post-vaccine effects can be obtained by monitoring changes in pediatric carriage.

## Conclusion

Our study shows that in a population of children aged from 6 to 24 months with a high rate of PCV7 vaccination coverage, antibiotic exposure, DCC attendance and AOM are linked to 19A carriage.

## Competing interests

Bernard Fritzell and Eric Bonnet are employed by Pfizer, and financial support was provided by Pfizer.

All the other authors declare that they have no competing interests.

## Authors' contributions

RC designed the study, prepared the study protocol and drafted the manuscript. CL coordinated the participating centers, was responsible for the statistical analysis and drafted the manuscript. EB and BF helped to draft the manuscript. FT and VD were the main investigators and helped to draft the manuscript. MB designed the database and helped to draft the manuscript. EB and EV were responsible for bacteriological analyses and helped to draft the manuscript. All the authors read and approved the final manuscript.

## Authors' information

Robert Cohen is a French pediatric infectious disease specialist, scientific director of a research institute on pediatric community acquired infections (ACTIV) and scientific director of a vaccine network for healthcare workers (Infovac-France). His main research interests are epidemiologic studies and clinical trials in community acquired infections including pneumococcal diseases, the rhinopharyngeal flora, and vaccines. He has published more than 40 papers in English language in these fields.

## Pre-publication history

The pre-publication history for this paper can be accessed here:

http://www.biomedcentral.com/1471-2334/11/95/prepub
